# Soleus subtendon elongation is reduced by individual suturing of Achilles subtendons after rupture

**DOI:** 10.1002/ksa.70533

**Published:** 2026-07-18

**Authors:** Rasmus Kramer Mikkelsen, Lars Konradsen, Rikke Hoeffner, Michael Rindom Krogsgaard, Anne‐Sofie Agergaard, Michael Kjaer, Rene Brueggebusch Svensson, Stig Peter Magnusson

**Affiliations:** ^1^ Section for Sports Traumatology, Department of Orthopedic Surgery Copenhagen University Hospital Bispebjerg‐Frederiksberg Copenhagen Denmark; ^2^ Department of Physical and Occupational Therapy Copenhagen University Hospital Bispebjerg‐Frederiksberg Copenhagen Denmark; ^3^ Institute of Sports Medicine Copenhagen, Department of Orthopaedic Surgery Copenhagen University Hospital Bispebjerg‐Frederiksberg Copenhagen Denmark; ^4^ Department of Clinical Medicine University of Copenhagen Copenhagen Denmark

**Keywords:** Achilles tendon, declarations, gastrocnemius, soleus, surgery, tendon length

## Abstract

**Purpose:**

The primary aim was to evaluate if individual suturing of the gastrocnemius and soleus parts of the ruptured Achilles tendon could reestablish their length 1 week post‐surgery compared to previously published results using a standard operative technique. We hypothesised that this new suturing technique would reduce the soleus subtendon length by 50% compared to the standard technique 1 week after surgery (primary endpoint). Secondary aims included soleus and gastrocnemius subtendon lengths at 26 weeks.

**Methods:**

The ambi‐directional design included a retrospective part using a standard Kessler repair of the tendon as a single structure (*n* = 24) and a prospective part using a novel surgery that sutured the soleus and gastrocnemius subtendons separately (*n* = 23). One and 26 weeks after surgery, both groups underwent 3D MRI to determine the length of the soleus and gastrocnemius subtendons.

**Results:**

For the soleus subtendon, the individual suturing technique yielded a smaller side‐to‐side (injured‐uninjured) difference (1.9 ± 14.7 mm) compared to the standard surgery (17.0 ± 11.5 mm, *p* < 0.0001) at week 1 and week 26 (individual 10.7 ± 14.9 mm, standard 21.5 ± 12.7 mm, *p* < 0.0001), although there was an increase in the individual suturing from week 1 to week 26, (*p* < 0.05). The side‐to‐side difference in gastrocnemius subtendon length was not significantly affected by suturing technique but increased with time for individual suturing from week 1 (−1.5 ± 10.6 mm) to week 26 (9.2 ± 9.4 mm, *p* < 0.0001), and for standard suturing from week 1 (1.8 ± 11.1 mm) to week 26 (8.4 ± 10.9 mm, *p* < 0.0001).

**Conclusion:**

Using an individual suturing approach, soleus and gastrocnemius subtendon lengths were nearly restored to normal values 1 week postoperatively. Subsequent elongation of the soleus subtendon over 6 months was also significantly less with individual suturing than standard surgical repair.

**Level of Evidence:**

Level III.

## Graphical abstract

The findings supported the hypothesis that individually suturing soleus and gastrocnemius subtendons reduces soleus subtendon elongation by approximately 50% compared with the standard technique 1 week after Achilles tendon repair. Although both subtendons elongated over 6 months, soleus elongation remained significantly lower with individual suturing, without affecting muscle size, patient‐reported outcomes, or heel‐rise performance.

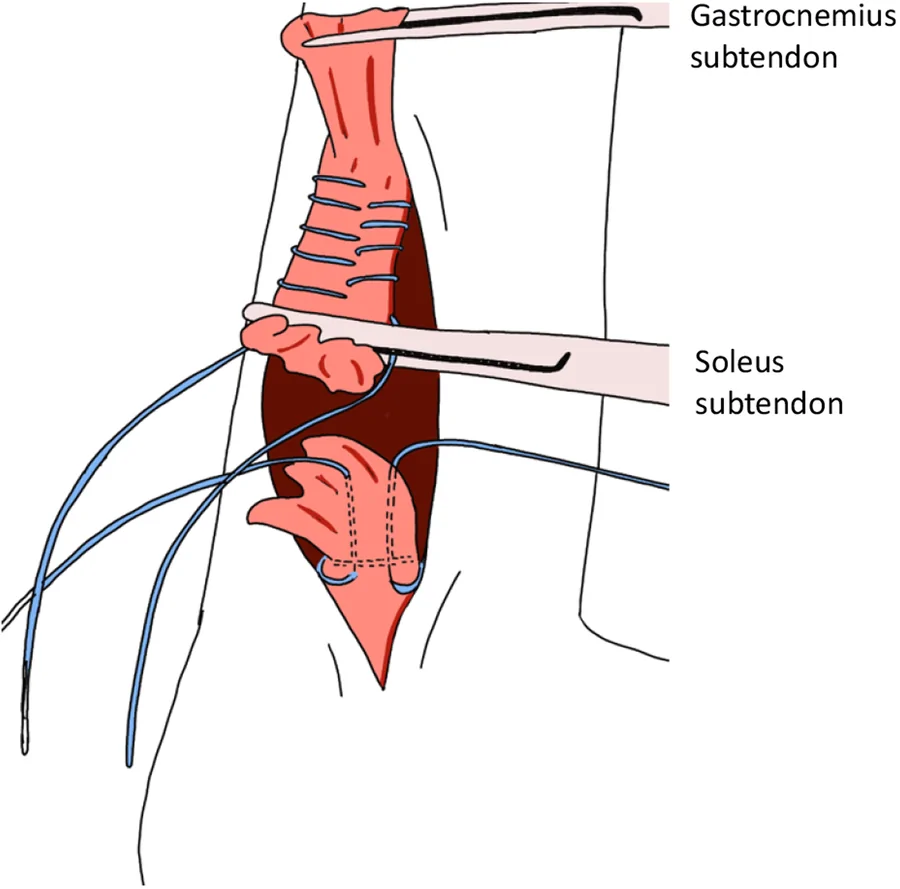

**Abbreviations**
ATRS: Achilles tendon total rupture score
CSA: cross‐sectional area
MRI: magnetic resonance imaging
PROM: patient‐reported outcome measures

## INTRODUCTION

Acute Achilles tendon rupture is a common injury that occurs most frequently in 30–50‐year‐old men participating in recreational sports [21, 27, 30]. The incidence has been documented to be approximately 18 cases per 100,000 individuals annually and seems to be on the rise [21, 25, 27, 30]. Irrespective of treatment, these patients are often left with an elongated Achilles tendon, calf muscle atrophy and weakness, which results in a permanently reduced physical ability [3, 11, 16, 29]. Return to pre‐injury physical activity varies substantially with the patient population [15]. For recreationally active patients, only 4 in 10 return to their pre‐injury physical activity, and only one in two patients find their symptom level to be acceptable [7, 19].

The best way to treat the Achilles tendon rupture remains an enigma and both surgical and non‐surgical treatment of acute Achilles tendon rupture frequently leave the patient with persistent functional deficit [3, 16, 29, 42]. A meta‐analysis demonstrated that the difference in re‐rupture rate and risk of complications between surgical and non‐surgical treatment is relatively small (risk difference 1.6% and 3.3%, respectively) [34]. Moreover, a recent large randomised controlled trial concluded that surgical and non‐surgical treatment yielded similar outcomes at 12 months; however, both treatments resulted in ∼20%–30% functional deficits [29], which highlights the need for improvement in treatment strategy. The reason for the lack of treatment progress remains elusive.

Efforts to improve surgery have largely focused on suturing techniques [9, 46]. The vast majority of these techniques have a failure load of less than 500 N and very few reach 800 N [9, 46], which is still well below the 1000–2000 N of tensile force in the Achilles tendon during regular ambulation [12]. The aim of suturing should therefore likely not be to resist tensile forces but enable healing of the original anatomy before the patient starts loading the tissue. Importantly, all suturing techniques have considered the tendon as a single structural unit. However, over a century ago it was recognised that the Achilles tendon has sub‐units that seem to be independent and ‘lay parallel to one another’ [36], and that there is a separate gastrocnemius and soleus portions of the free Achilles tendon below the most distal soleus muscle fibres [8]. While the gastrocnemius and soleus portions merge to form the free Achilles tendon, it is well established that they can be considered functionally separate musculotendinous units [1, 5, 13], however, this is not routinely addressed during surgical repair of the Achilles tendon.

Using a 3D magnetic resonance imaging (MRI) technique, it was recently shown that already 1 week after surgical repair of the Achilles tendon without any prior weightbearing, the soleus portion of the Achilles tendon was 35% longer but that the gastrocnemius portion was merely 6% longer compared to the contralateral uninjured side [18]. This marked length discrepancy between the gastrocnemius and soleus subtendons of the free Achilles tendon shortly after surgery implies that surgery itself did not reestablish the anatomy of the Achilles tendon. Inspired by these novel and previously unrecognised observations in combination with earlier described surgery techniques [38, 39, 45], we have adopted an Achilles tendon surgical repair technique with special emphasis on normalising the length of the soleus compared to the uninjured contralateral side per‐operatively. The tendon elongates in the months after surgery and because this elongation is affected by various other factors such as rehabilitation, the isolated impact of a given surgery on the Achilles subtendon lengths must be evaluated shortly after surgery and prior to any loading [11, 18, 28, 35].

The primary aim of the present study was to evaluate if individual suturing of the two parts of the ruptured Achilles tendon could reestablish the length of the soleus and gastrocnemius parts of the tendon 1 week post‐surgery compared to previously published results using a standard operative technique. We hypothesised that the new individual suturing technique would reduce the elongation of the soleus subtendon (primary outcome) of the Achilles tendon to 9 mm or less (~50% reduction from the 19 mm difference previously observed between injured and uninjured with the standard technique at week 1 [18] when compared to the standard technique 1 week after surgery (primary endpoint). The secondary aims were to evaluate the effect of this new technique on soleus and gastrocnemius subtendon lengths (26 weeks), muscle morphology (26 and 52 weeks) and functional (26 and 52 weeks) and clinical outcomes (52 weeks).

## MATERIALS AND METHODS

### Study design

Approval was obtained from the regional ethics committee for the prospective (No. H‐22033710) and retrospective part (No. H‐19034916), and from the Danish Data Protection Agency. All participants received oral and written information prior to providing their written informed consent.

This was an ambi‐directional study that included prospective (ClinicalTrials.gov NCT05717270) and retrospective data (ClinicalTrials.gov NCT04263493) [18]. The retrospective data have been reported in detail previously [18]. The participants of the retrospective part underwent a standard operating procedure (see below) and received the current standard rehabilitation (*n* = 24). The prospective participants (*n* = 23) underwent a new surgical procedure (see below) followed by the same rehabilitation program as the retrospective participants. The following data were collected: MRI (weeks 1 and 26), heel‐rise test (weeks 26 and 52) and Achilles tendon total rupture score (ATRS, week 52).

### Participants

The patient recruitment was achieved in‐house from the emergency clinic and the orthopaedic surgery department at Copenhagen University Hospital Bispebjerg and Frederiksberg. Potential participants were identified through screening of the orthopaedic surgery lists. When the diagnosis of Achilles tendon rupture was established, patients were orally informed about the study and if interested in participating, they received the written information. Inclusion criteria were; diagnosed with a traumatic complete mid‐substance Achilles tendon rupture within 14 days from injury and no contraindication for MRI, age 18–60 years, understanding Danish, and managing transportation to/from the hospital on their own. Exclusion criteria were; other injuries affecting the lower limb functions, prior contralateral Achilles tendon rupture, re‐rupture, smoking, systemic diseases influencing tendon healing, and anticoagulation treatment. We allowed up to 14 days from injury to surgery because it has been reported that surgical outcomes do not differ within this period [6].

### Suturing techniques

The standard surgical technique used in the retrospective part has been described earlier [18]. Briefly, experienced surgeons performed open repair under local anaesthesia using the Kessler technique while the patients were lying prone with their feet hanging freely at the end of the operating table. Sutures were tightened until the ankle resting angle with fully extended knee was the same as on the uninjured side. A below‐knee plaster cast with the ankle in 30° of plantar flexion was applied to the lower limb.

The technique of individual subtendon suturing was based on the modified lace technique originally described by Segesser and Weisskopf [45] with further modifications as reported by others [38, 39]. Ultrasound imaging was used to identify the distance from the most superior corner of the calcaneus to the most distal muscle fibre of the soleus on the uninjured side (Figure 1a–c). This distance was then used as the new target length on the injured side. The patient was placed in the prone position under general or spinal anaesthesia to ensure complete relaxation of the lower limb muscles. The lower extremity was prepped and draped in a sterile manner from just below the knee to the midfoot. A posteromedial skin incision was made and the paratenon was incised longitudinally. The proximal part of the rupture was flipped around to define the distal muscle fibres of the soleus and the soleus/gastrocnemius tendon separation. The separation between the soleus and the gastrocnemius was identified, and the two subtendon units were separated. There was some heterogeneity in anatomy, sometimes easily defined and sometimes less distinct [40]. The deep layer (soleus) was separated from the superficial layer (gastrocnemius) (Figure 2a). Careful and thorough mobilisation was performed proximally using a Cobbs elevator to free the tendon and soleus muscle on the deep side. Substantial adhesions were present already 1 week after injury. Breaking the adhesions with a Cobbs elevator was necessary to be able to pull the proximal stump of the soleus sufficiently distally. Using Fiberwire 2®, whip stich sutures were placed in the proximal soleus subtendon stump and Kessler sutures deep in the distal stump (Figure 2b). The distal muscle fibres of the soleus were inspected, and intraoperatively the distance from the very distal soleus muscle fibres to the top of the calcaneus was measured and the tendon pulled distally until reaching the distance previously measured on the uninjured contralateral side. Fiberwire 2® whip stitch sutures were placed in the gastrocnemius aponeurosis proximally and Kessler sutures superficially in the distal tendon stump (Figure 3a,b). Reestablishing the soleus subtendon length resulted in an exaggerated degree of plantarflexion as compared to the uninjured side. The tightness was checked with the Mattles test. Finally, the wound was closed using nylon Matress sutures, and a below‐knee plaster cast with the ankle in 30° of plantar flexion was applied to the lower limb. Note that while the primary distinction between the surgical techniques is the individual suturing, the nature of the surgery necessitates that other aspects also differ, including the length of the incision, level of anaesthesia, perioperative degree of plantarflexion and possibly other factors. The comparison is therefore between the entire surgical techniques as described here.

**Figure 1:**
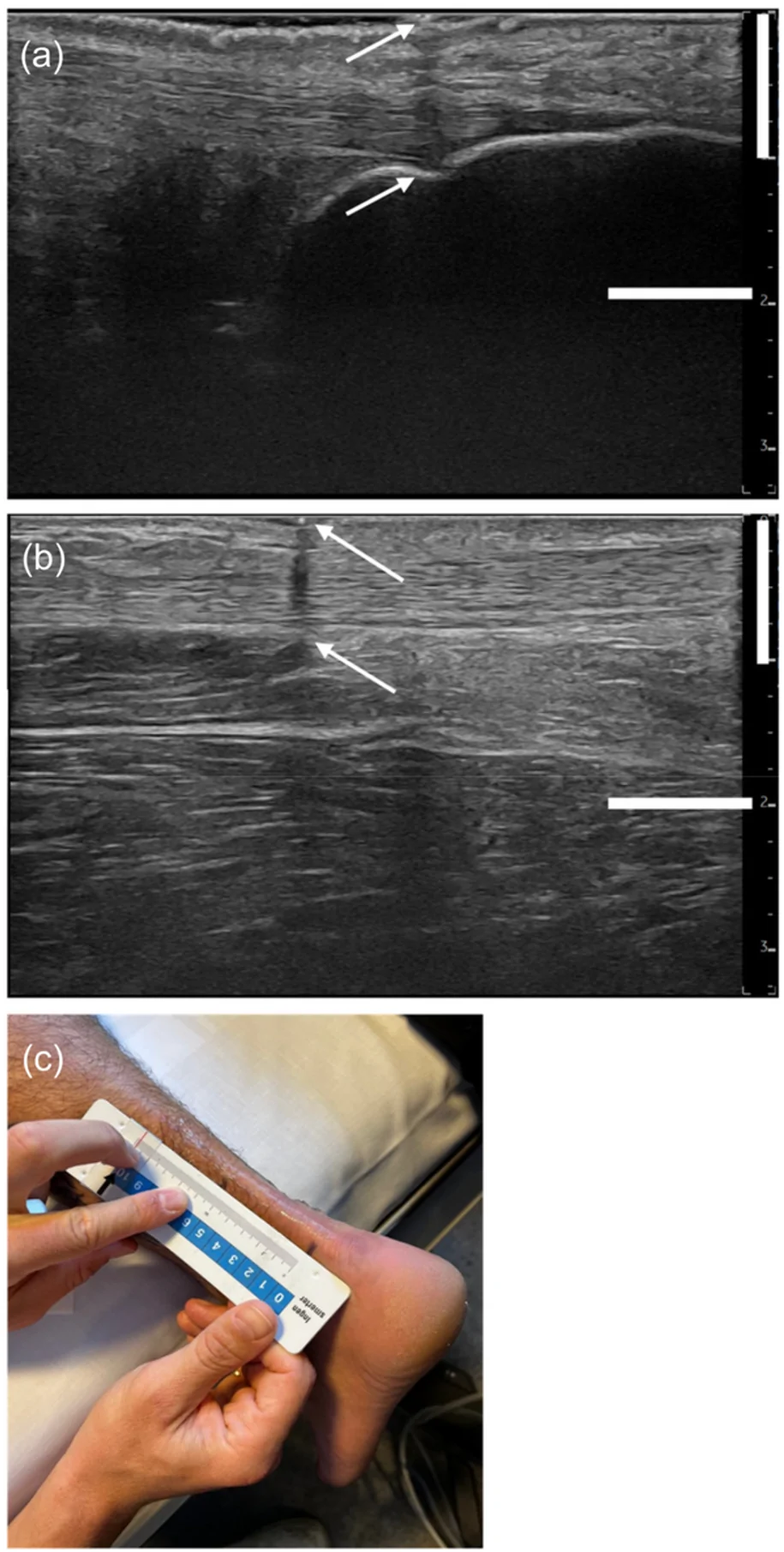
(a) Ultrasound measurement of the proximal corner of the calcaneus on the contralateral healthy Achilles tendon. Top arrow: needle creating shadow on calcaneus. Bottom arrow: proximal corner of the calcaneus. Horizontal and vertical bars indicate 10 mm. (b) Ultrasound measurement of the most distal soleus muscle fibre on the contralateral healthy Achilles tendon. Top arrow: needle creating shadow on distal muscle fibres. Bottom arrow: most distal soleus muscle fibre. Horizontal and vertical bars indicate 10 mm. (c) The external markers based on the identified landmarks using ultrasound in (a) and (b).

**Figure 2:**
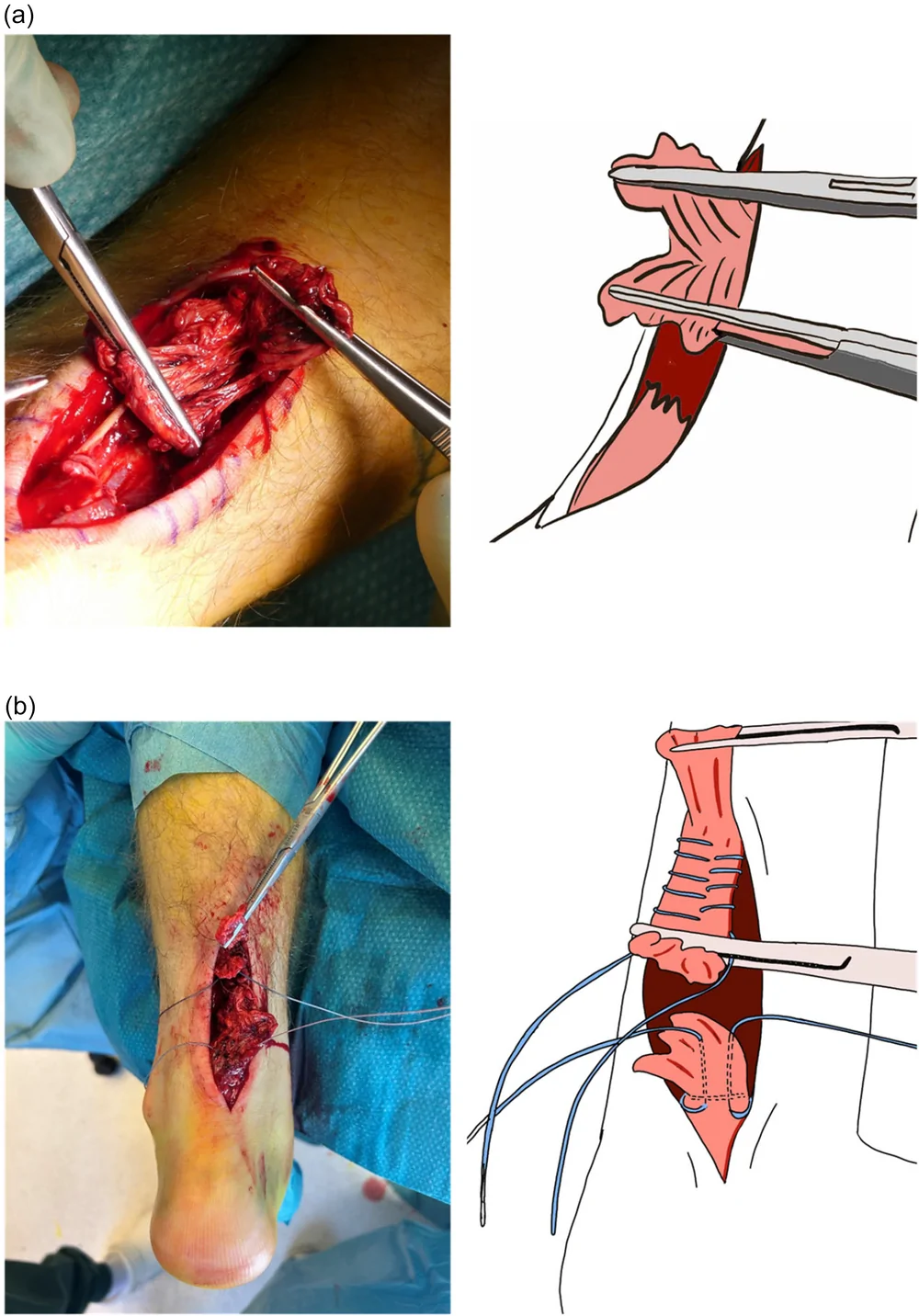
(a) The proximal Achilles tendon stump has been dissected into a superficial gastrocnemius subtendon and a deep soleus subtendon. (b) Whip stich suture in the proximal and Kessler suture in the distal stump of the soleus subtendon.

**Figure 3:**
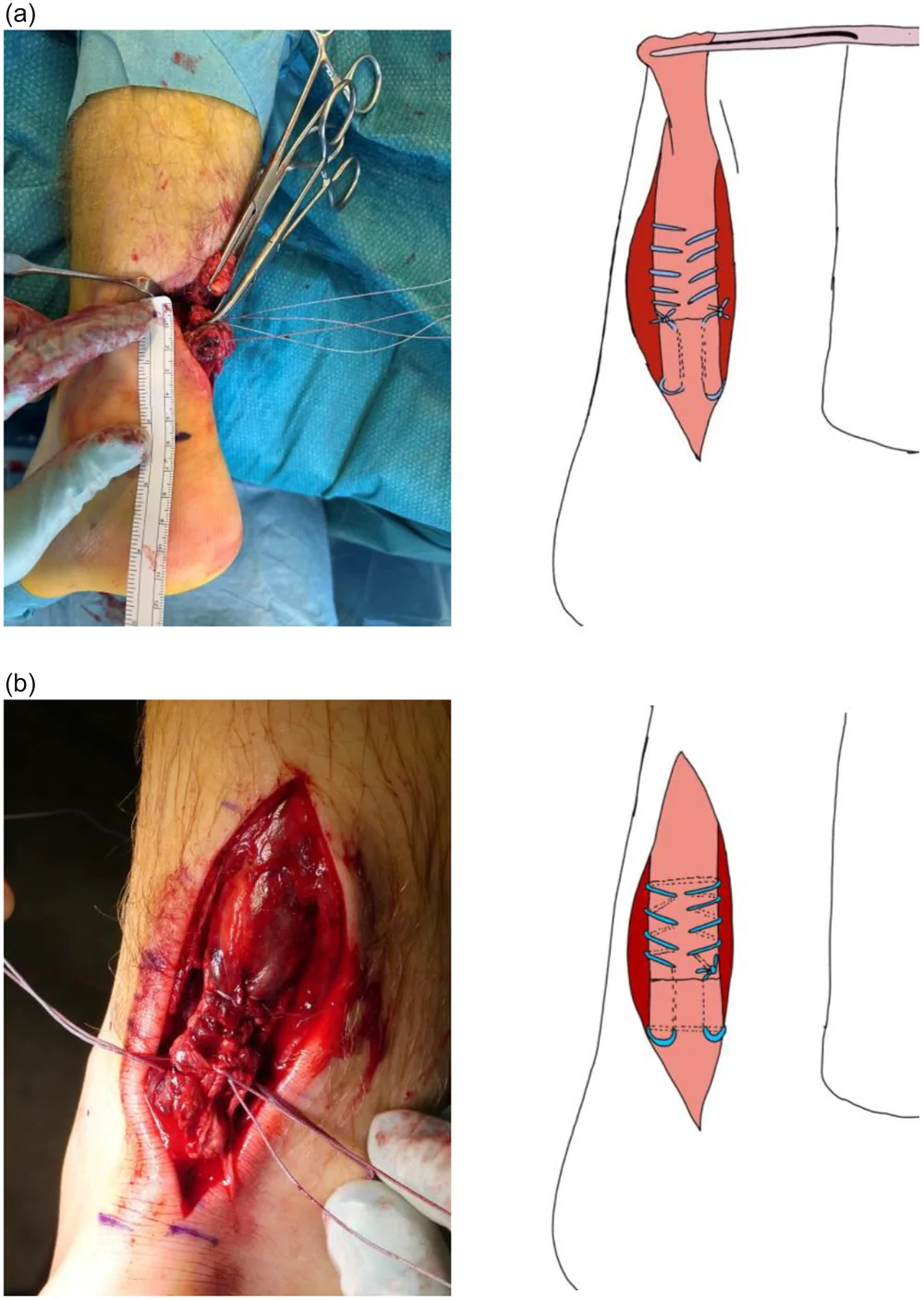
(a) The sutured Soleus subtendon approximated to the normal calcaneus‐distal soleus fibre length as was measured on the healthy contralateral side. (b) The gastrocnemius subtendon sutured to the distal stump under tension.

To optimise and align the surgical technique, the two surgeons that participated in the prospective part initially performed five surgeries together. After each surgery an MRI was obtained and the length of the soleus and gastrocnemius subtendons were measured and reported back to the operating surgeons as feedback for optimising the procedure.

### Rehabilitation

The rehabilitation for the participants in the retrospective part has been published previously [18].

The rehabilitation for the prospective study was identical to that of the control group in the retrospective part. In brief, no weightbearing was allowed on the plaster cast, which was worn for 2 weeks. At week 2, the cast and sutures were removed, and an orthosis was used with partial weight bearing. Patients removed the orthosis to perform active ankle joint range of motion exercises five times a day. On week 7 the orthosis was removed and full weightbearing was allowed, followed by progressively increased loading activities, with jogging allowed at 6 months and return to contact sports 8–9 months after surgery.

### Outcome measures

The outcomes of the present study were measured identically to the retrospective part [18]. The primary outcome was soleus subtendon length difference between the injured and uninjured side 1 week after surgery. Secondary outcomes were; (a) soleus subtendon length difference between the injured and uninjured side at 26 weeks, (b) gastrocnemius subtendon length difference between the injured and uninjured side at week 1 and 26, (c) heel‐rise height, repetitions and total work at week 26 and 52, (d) muscle cross sectional area of the soleus and gastrocnemii at week 1 and 26, and (e) ATRS at 52 weeks.

Tendon length and muscle cross‐sectional area (CSA) were measured by MRI on a 1.5T scanner (Ingenia Ambition, Philips). For the tendon length measurements, three landmark points in 3D were defined at the medial gastrocnemius and soleus muscle distal insertions and the calcaneus proximal insertion as previously outlined [43]. The CSA of the soleus, medial and lateral gastrocnemius muscles were measured manually at one third of the length of the triceps surae muscle. Analyses for the prospective part were blinded for side and the retrospective data.

Heel‐rise muscle function and endurance were evaluated using a standardised heel‐rise test [41, 42]. In brief, the patient was standing on a box with the ankle in neutral position and the heel‐rise test was performed barefooted on one leg at a time and at a cadence at 30 rises per minute.

Patient symptoms, function and treatment satisfaction were evaluated using the Danish version of the ATRS [32], which has been validated using classical test theory [14].

### Statistical analysis

The sample size for the prospective part was based on the soleus subtendon lengthening 1 week after standard surgery across all participants previously published [18]. The study was designed to observe a 50% reduction in soleus subtendon elongation (primary outcome). The numbers used were; *u*_A_ = 19 mm, *u*_B_ = 9 mm, σ = 17 and a power of 80% resulting in a sample size of *n* = 23.

Difference values refer to injured minus uninjured side in the outcome measures unless otherwise noted. Q–Q plots were visually examined for distribution normality. All data were analysed using GraphPad Prism 10.5.0. The data are shown as mean ± SD in tables and ± 95% confidence interval in figures. Mixed‐effects analyses (group x time) were used to analyse tendon length, muscle CSA, and heel‐rise outcome parameters. Sidaks test with correction for multiple comparisons were used for post hoc analyses. A two‐tailed unpaired *t*‐test was used to compare ATRS data. The significant level for all tests was set to *p* ≤ 0.05.

## RESULTS

The baseline characteristics of the patients are presented in Table 1.

**Table 1:** Descriptive statistics for the groups.

Characteristics	Standard suturing (*n* = 24)	Individual suturing (*n* = 23)
Male sex, *n* (%)	17 (71%)	18 (75%)
Age, years	36 ± 10	37 ± 9
Height, cm	179 ± 8	179 ± 11
Body mass index, kg/m^2^	25 ± 3	26 ± 3
Injured side right/left, *n*	10/14	12/12

*Note*: Data are presented as count (%) for sex data and mean (SD) for continuous data.

### Adverse events and complications

In the retrospective part, there were two cases of deep infection, one case of deep vein thrombosis, and two patients reported neuropathic symptoms, which had fully resolved by 52 weeks [18]. In the prospective part, three participants presented with incompletely healed midline scars at 2 weeks. Two of them had no signs of infection and just retained sutures for an additional week. The third had signs of superficial infection and received treatment with a 1‐week course of Dicillin and application of a PICO pump. One participant sustained an Achilles tendon rupture on the contralateral side 12 months postoperatively and did therefore not perform the heel‐rise test at 52 weeks.

### Subtendon length

Subtendon length data are shown in Figure 4a,b. There was a significant time (*p* < 0.001), and group (*p* < 0.001), but no interaction effect (*p* = 0.266) for soleus subtendon length. The individual surgery technique yielded a significantly smaller side‐to‐side difference (1.9 ± 14.7 mm) compared to the standard surgery technique (17.0 ± 11.5 mm, *p* < 0.001) at week 1 and week 26 (individual 10.7 ± 14.9 mm, standard 21.5 ± 12.7 mm, *p* < 0.05). There was a significant increase in side‐to‐side difference with the individual suturing from week 1 to week 26, (*p* < 0.01).

**Figure 4:**
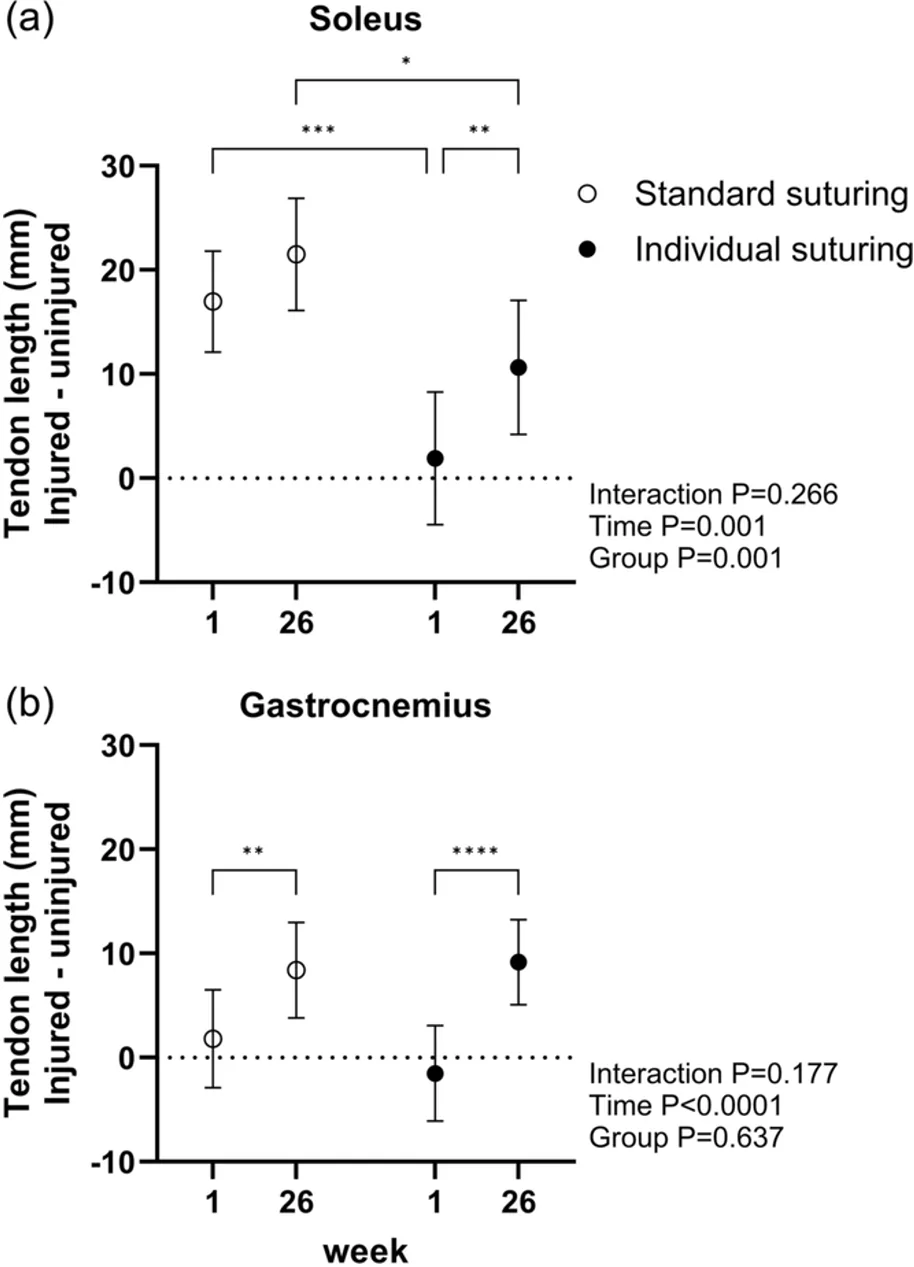
The subtendon length difference between the injured and uninjured side for the standard and individual suturing technique. (a) The soleus subtendon length increased from week 1 to 26 with the individual suturing technique (**p < 0.01) and resulted in a smaller side‐to‐side difference compared to the standard suturing technique at week 1 (***p < 0.0001) and week 26 (*p < 0.05). (b) The gastrocnemius subtendon length increased with time for both suturing techniques (****p < 0.0001). Data are presented as mean ± 95% confidence interval.

There was no significant group or interaction effect but a significant effect of time (*p* < 0.0001) for gastrocnemius subtendon length. The side‐to‐side length difference increased with time for individual suturing from week 1 (−1.5 ± 10.6 mm) to week 26 (9.2 ± 9.4 mm, *p* < 0.0001). It also increased with time for standard suturing from week 1 (1.8 ± 11.1 mm) to week 26 (8.4 ± 10.9 mm, *p* < 0.01). The absolute values for subtendon length are shown in the supplement data (Supporting Information S1: Table S1).

### Muscle morphology

Muscle CSA data are shown in Figure 5a–c. For the soleus muscle, there was a significant time effect (*p* < 0.0001), but no group or interaction effect. Across groups, the side‐to‐side difference in CSA declined over time from week 1 (−2.2 ± 3.7 cm^2^) to week 26 (−6.3 ± 3.4 cm^2^ *p* < 0.0001). There was no significant time, group or interaction effect for the medial gastrocnemius muscle CSA, but for the lateral there was a significant effect of time (*p* < 0.05). Across groups, the side‐to‐side difference in CSA increased over time from week 1 (−1.6 ± 2.2 cm^2^) to week 26 (−0.7 ± 1.9 cm^2^, *p* < 0.05). The absolute values for muscle CSA are shown in the supplement data (Supporting Information S1: Table S2).

**Figure 5:**
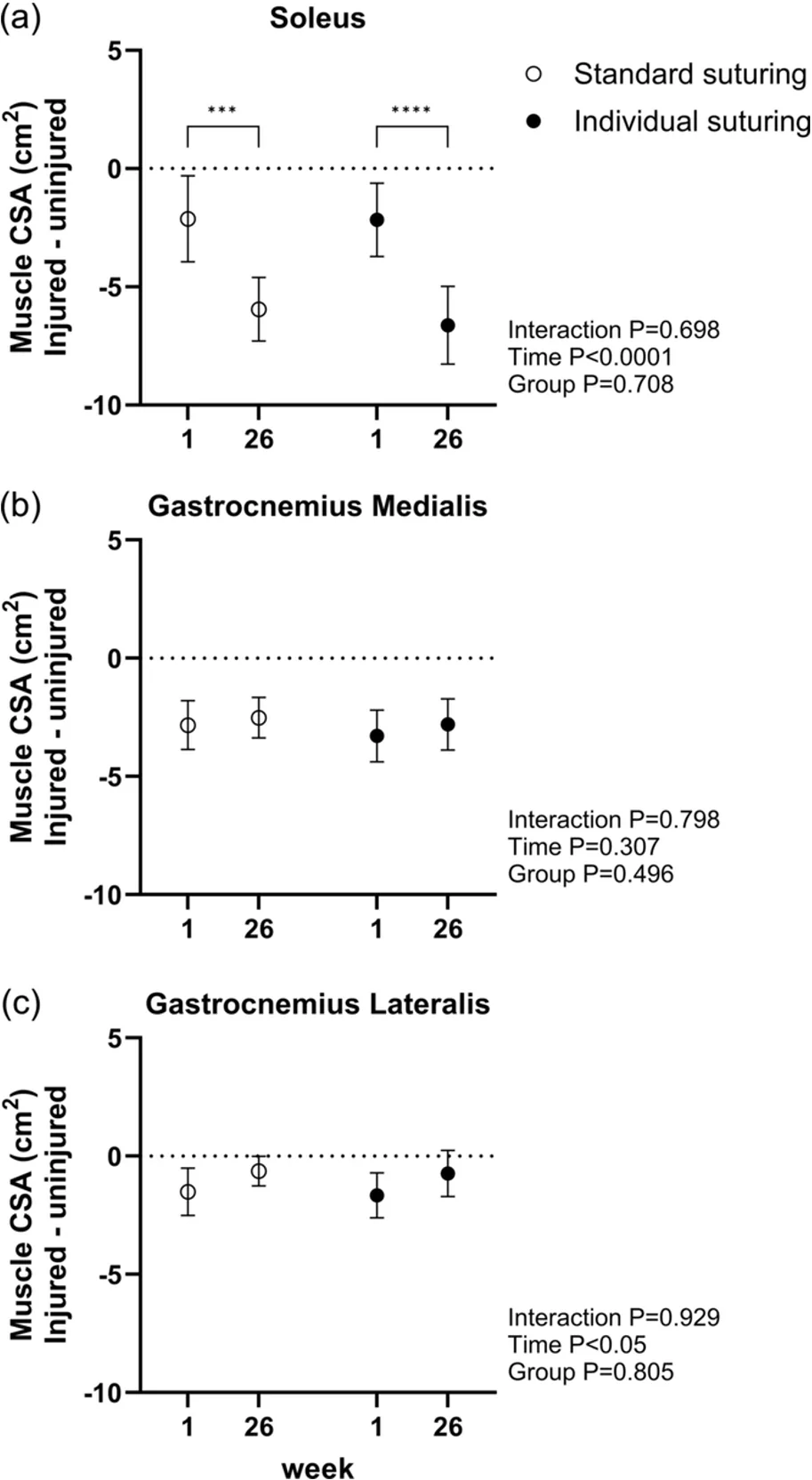
The muscle cross‐sectional area (CSA) difference between the injured and uninjured side for the standard and individual suturing technique. (a) The soleus muscle CSA decreased from week 1 to 26 with both suturing techniques (***p < 0.001, ****p < 0.0001). (b) The medial gastrocnemius muscle displayed atrophy at week 1 that persisted to week 26 with both suturing techniques. (c) The lateral gastrocnemius muscle displayed atrophy at week 1 which was reduced at week 26 with both suturing techniques. Data are presented as mean ± 95% confidence interval.

### Heel‐rise test

Heel‐rise test results are shown in Figure 6a–c. Deficits in heel‐rise height, repetitions and work displayed a significant effect of time (*p* < 0.0001), but no group or interaction effect. Across groups, there was an increase over time for; heel rise height from week 26 (−3.5 ± 2.0 cm) to week 52 (−2.1 ± 1.3 cm, *p* < 0.0001), heel‐rise repetitions from week 26 (−9.7 ± 8.6) to week 52 (−3.6 ± 7.5, *p* < 0.0001) and heel‐rise work from week 26 (−1136 ± 690 J) to week 52 (−592 ± 596 J, *p* < 0.0001). The absolute values for heel‐rise outcomes are shown in the supplement data (Supporting Information S1: Table S3).

**Figure 6:**
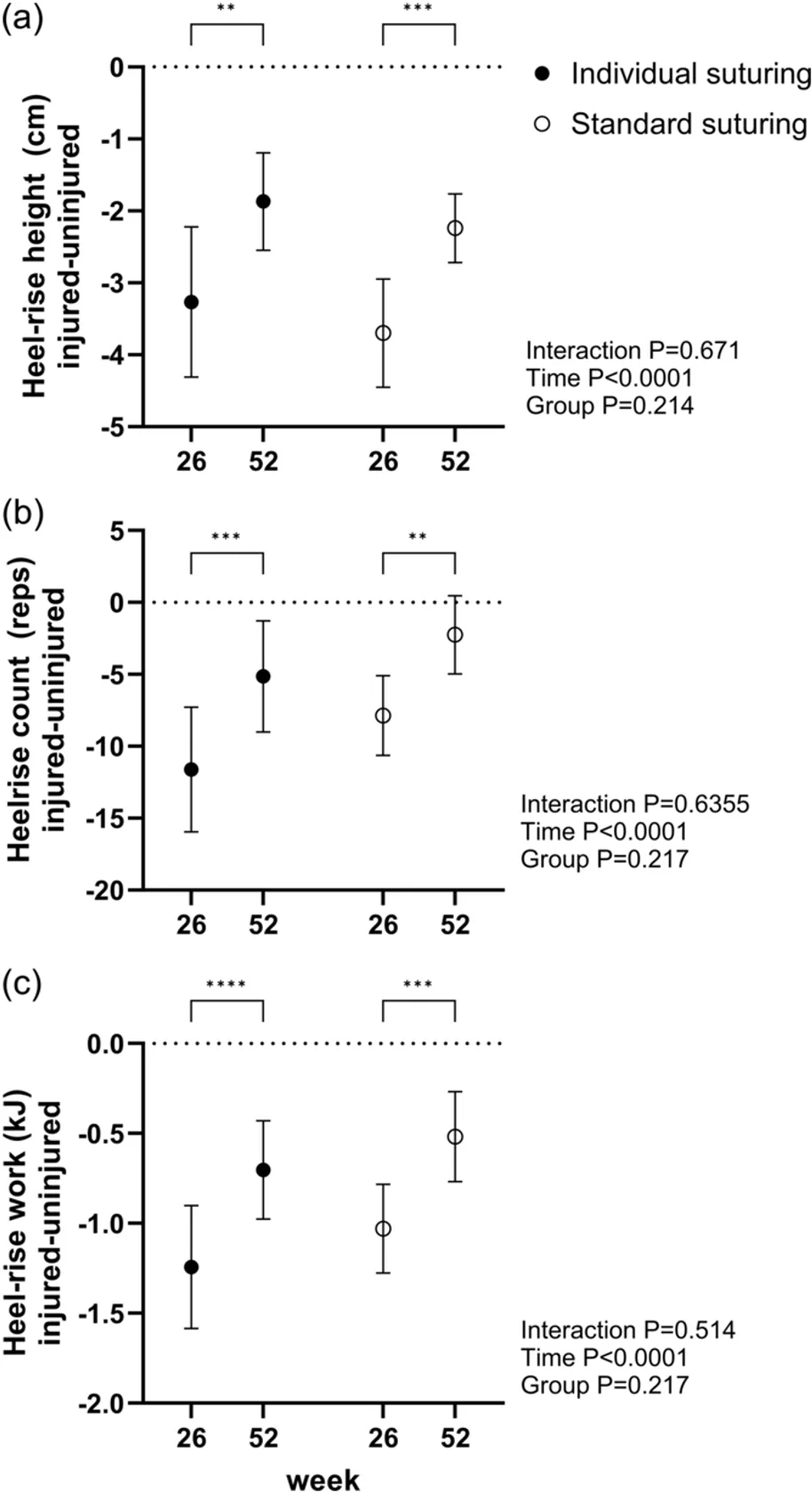
Heel‐rise performance. (a) Height, (b) repetitions and (c) work. Significantly different from 26 weeks (**p < 0.01, ***p < 0.001, ****p < 0.0001).

### Patient‐reported outcome measure

There was no significant difference between standard suturing (59.6 ± 22.1) and individual suturing (70.2 ± 19.6) in the ATRS (*p* = 0.09).

## DISCUSSION

The results suggest that the individual suturing technique was able to restore soleus subtendon length 1 week after surgery, that is, it was possible to avoid the early soleus muscle retraction that was previously observed for the standard suturing technique in Achilles tendon ruptures [18]. The gastrocnemius subtendon was restored by either surgical technique. Both subtendons elongated during the subsequent 6 months of rehabilitation, however, the soleus subtendon elongation was reduced by 50% with individual suturing compared to the standard technique at 6 months.

Although it has been acknowledged that the Achilles tendon has anatomically independent subtendons a long time ago [8, 36], it has not received a great deal of attention in the surgical treatment for Achilles tendon rupture. Consequently, most studies investigating Achilles tendon length after rupture have measured the gastrocnemius subtendon and may therefore have overlooked the possible distinct retraction and elongation of the soleus subtendon. Since sutures themselves cannot withstand the level of tensile force seen by the Achilles tendon during regular ambulation [12], the surgical treatment of the injury primarily serves to approximate the rupture ends to facilitate healing of the tendon tissue. Therefore, surgically re‐establishing the original anatomy may be important for the tendon to heal with minimal elongation, especially given the marked retraction of the soleus subtendon seen with standard suturing techniques [18]. The prospective data of the current study suggest that the anatomy of the free Achilles tendon with its subtendons can in fact be reestablished through the new surgical approach as evidenced by the 1‐week data.

Several studies have shown that the Achilles tendon elongates during the healing process [11, 18, 28], and that this takes place in the initial 6 month post‐surgically [11]. Moreover, it appears that this elongation can primarily be attributed to that of the gastrocnemius subtendon while the soleus subtendon is retracted and elongated already from the onset [18]. The current prospective data corroborates that the gastrocnemius subtendon elongates during the initial 6 months after surgery. The data also show that when the soleus subtendon length has been surgically reestablished, it elongates with a magnitude similar to the gastrocnemius during the first 6 months. Collectively, these data show the effect of surgery (week 1) and the combined effect of surgery and rehabilitation (week 26) on tendon length. It remains unclear exactly what contributes to this rehabilitation‐associated increase in tendon length, and how to mitigate it.

It has been shown that the CSA of the triceps surae muscles do not necessarily recover after an Achilles tendon rupture and may even have a permanent deficit [11, 16]. It is notable that persistent muscle atrophy is more pronounced in the soleus compared to the gastrocnemii muscles after Achilles tendon rupture [11, 16, 18]. The soleus muscle contributes approximately two‐thirds of the triceps surae physiological CSA [44], and therefore this atrophy likely impacts muscle force generation after an Achilles tendon rupture [18, 19]. Here, we show that all three parts of the triceps surae muscle display about a 2%–3% deficit 1 week after surgery. However, it is noteworthy that while the gastrocnemii retain or slightly recover their muscle mass, the deficit of the soleus muscle CSA is augmented 3‐fold over 6 months, even when soleus subtendon length is surgically reestablished. Whether the persistent loss of soleus muscle mass relates to its considerable accumulation of intramuscular fat [10, 18], an inability of contractile proteins to adjust to a shorter muscle fibre length because of tendon elongation [20], or other factors remains unknown.

The suturing technique did not influence the heel‐rise performance, which corroborates prior to studies that have not been able to improve on this outcome [2, 11, 18, 31, 33]. The heel‐rise height deficit may be more affected by tendon elongation coupled muscle fascicle shortening than by strength per se [4, 37, 42, 43], as muscle excursion relates to fibre length [23, 26]. Though heel‐rise height has been related to gastrocnemius tendon length [37, 42, 43], the relative contribution of soleus subtendon elongation and accompanying soleus shortening on heel‐rise height remains unknown.

The ATRS is a patient‐reported instrument that specifically assesses the manifestation of symptoms and the degree of physical activity following a complete rupture of the Achilles tendon [32]. The ATRS score for standard (59.6 ± 22.1) and individual suturing (70.2 ± 19.6) was not statistically different (*p* = 0.09). However, the difference may be clinically important since the clinically relevant difference has been suggested at 10‐points [32]. The clinical outcome has been shown to be linked to tendon elongation [17, 42, 43]. It is unclear why the surgical technique did not statistically affect the ATRS score but may relate to the elongation of the subtendons from week 1 to 26. Considering psychological factors in the therapeutic approach following this particular injury may also be important [22, 24].

A limitation of the present study is the ambi‐directional design using a combination of prospective and retrospective data rather than a prospectively randomised controlled trial, which should be undertaken. However, the prospective data was collected adjacent to the end of the retrospective data collection, and both were conducted at the same hospital, and thus the patient groups are likely comparable, which is supported by the characteristics (Table 1). The marked retraction of the soleus part of the tendon with the standard suturing technique made us question if our standard technique had been inadequately performed; however, we used the (standard) Kessler technique described in standard textbooks, and the surgery was performed by experienced orthopaedic surgeons with a special interest in Achilles tendon surgery. The fact that both the gastrocnemius and soleus subtendons elongated from week 1 to week 26 (Figure 4), and that it has been shown that it does not appears to elongate beyond 26 weeks [11, 18] prompts questions surrounding the load placed on the tendon. While we used an ordinary rehabilitation program previously published [18], the load was not carefully monitored throughout the rehabilitation period.

## CONCLUSION

The data supported the hypothesised 50% reduced elongation of the soleus part of the Achilles tendon when comparing individual suturing to the standard technique [18] 1 week after surgery. In fact, 1 week after surgery the soleus subtendon was nearly re‐established (1.9 mm side‐to‐side difference). Both the soleus and gastrocnemius subtendons elongated over 6 months; however, the soleus subtendon remained significantly less elongated with the individual suturing compared to the standard suturing. The individual suturing technique did not impact muscle CSA, the patient‐reported outcome or heel‐rise function.

## AUTHOR CONTRIBUTIONS

Conceptualisation: Developing the idea & research goals (Rasmus Kramer Mikkelsen, Lars Konradsen, Rikke Hoeffner, Michael Rindom Krogsgaard, Anne‐Sofie Agergaard, Rene Brueggebusch Svensson and Stig Peter Magnusson). Methodology: Designing methods used for data collection and analysis (Rasmus Kramer Mikkelsen, Lars Konradsen, Rikke Hoeffner, Rene Brueggebusch Svensson and Stig Peter Magnusson). Investigation: Performing experiments or collecting data (Rasmus Kramer Mikkelsen, Lars Konradsen, Rikke Hoeffner and Rene Brueggebusch Svensson). Data Curation: Managing and organising data for analysis (Rikke Hoeffner, Anne‐Sofie Agergaard, Rene Brueggebusch Svensson and Stig Peter Magnusson). Formal Analysis: Performing statistical or computational analysis (Rikke Hoeffner, Michael Rindom Krogsgaard, Rene Brueggebusch Svensson and Stig Peter Magnusson). Writing—Original Draft: Preparing initial manuscript (Rasmus Kramer Mikkelsen, Stig Peter Magnusson). Writing—Review and Editing: Revising the manuscript (Rasmus Kramer Mikkelsen, Lars Konradsen, Rikke Hoeffner, Michael Rindom Krogsgaard, Anne‐Sofie Agergaard, Rene Brueggebusch Svensson and Stig Peter Magnusson). Visualisation: Creating figures & graphs (Rasmus Kramer Mikkelsen, Lars Konradsen, Michael Rindom Krogsgaard and Stig Peter Magnusson).

## CONFLICT OF INTEREST STATEMENT

The authors declare no conflicts of interest.

## ETHICS STATEMENT

Approval was obtained from the regional ethics committee for the prospective (No. H‐22033710) and retrospective part (No. H‐19034916). All participants were given oral and written information about the study and gave written informed consent.

## Supporting information

Supporting File 1

## Data Availability

The data that support the findings of this study are available from the corresponding author upon reasonable request.
