# Supplementary material for: Soleus subtendon elongation is reduced by individual suturing of Achilles subtendons after rupture
Source: Knee Surg Sports Traumatol Arthrosc. 2026 Jul 18;34(10):3783–93. doi: 10.1002/ksa.70533 (PMC13622908; doi:10.1002/ksa.70533)
Supplement: Supplementary file 1 [file KSA-34-3783-s001.docx]

**TABLE S1**

|  | **Individual suture** | | **Standard suture** | |
| --- | --- | --- | --- | --- |
|  | Week | | Week | |
| **Tendon length (mm)** | 1 | 26 | 1 | 26 |
| **Gastrocnemius** |  |  |  |  |
| Uninjured | 185.2±22.1 | 186.0±22.1 | 187.9±19.3 | 189.6±18.9 |
| Injured | 183.7±25.9 | 195.1±21.5 | 189.7±23.5 | 198.0±16.6 |
| Difference | -1.5±10.6 | 9.2±9.4 | 1.8±11.1 | 8.4.±10.9 |
|  |  |  |  |  |
| **Soleus** |  |  |  |  |
| Uninjured | 56.0±18.8 | 56.4±19.9 | 51.9±15.0 | 53.3±16.2 |
| Injured | 57.9±20.9 | 67.1±17.8 | 68.8±18.4 | 74.8±17.5 |
| Difference | 1.9±14.7 | 10.7±14.9 | 17.0±11.5 | 21.5±12.8 |

Mean±SD

**TABLE S2**

|  | **Individual suture** | | **Standard suture** | |
| --- | --- | --- | --- | --- |
|  | Week | | Week | |
| **Muscle CSA (cm^2^)** | 1 | 26 | 1 | 26 |
| **M. gastrocnemius** |  |  |  |  |
| Uninjured | 15.4±3.7 | 15.4±4.1 | 15.1±4.0 | 15.1±3.9 |
| Injured | 12.1±3.5 | 12.6±3.6 | 12.7±3.8 | 13.0±3.7 |
| Difference | -3.3±2.5 | 2.8±2.5 | -2.8±2.3 | -2.5±1.8 |
|  |  |  |  |  |
| **L. gastrocnemius** |  |  |  |  |
| Uninjured | 6.8±2.6 | 7.3±3.3 | 7.0±3.0 | 6.7±2.7 |
| Injured | 5.2±2.8 | 6.5±2.9 | 5.5±2.2 | 6.2±2.3 |
| Difference | -1.7±2.2 | -0.7±2.3 | -1.4±2.2 | -0.6±1.3 |
|  |  |  |  |  |
| **Soleus** |  |  |  |  |
| Uninjured | 29.4±5.2 | 29.4±5.8 | 28.9±6.8 | 29.7±6.0 |
| Injured | 27.2±5.7 | 22.7±4.5 | 27.7±8.3 | 24.2±5.4 |
| Difference | -2.2±3.6 | -6.6±3.8 | -2.1±4.0 | -6.0±2.9 |

Mean±SD

**TABLE S3**

|  | **Individual suture** | | **Standard suture** | |
| --- | --- | --- | --- | --- |
|  | Week | | Week | |
| **Heel-rise** | 26 | 52 | 26 | 52 |
| **Height (cm)** |  |  |  |  |
| Uninjured | 11.9±1.4 | 11.8±1.5 | 11.9±1.4 | 11.8±1.5 |
| Injured | 8.6±2.2 | 9.9±1.8 | 8.6±2.2 | 9.9±1.8 |
| Difference | -3.3±2.3 | -1.8±1.4 | -3.3±2.3 | -1.8±1.4 |
|  |  |  |  |  |
| **Repetitions (#)** |  |  |  |  |
| Uninjured | 30.4±12.1 | 30.9±12.6 | 30.4±12.1 | 30.9±12.6 |
| Injured | 18.7±7.1 | 25.8±9.7 | 18.7±7.1 | 25.8±9.7 |
| Difference | -11.7±10.1 | -5.1±8.5 | -11.7±10.1 | -5.1±8.5 |
|  |  |  |  |  |
| **Work (J)** |  |  |  |  |
| Uninjured | 2136±921 | 2244±790 | 2197±893 | 2244±790 |
| Injured | 942±661 | 1541±616 | 954±453 | 1541±616 |
| Difference | -1150±819 | -703±600 | -1242±791 | -703±600 |

Mean±SD
